# Post-dural Puncture Headache After Removal of Trail Spinal Cord Stimulator Leads: A Case Report

**DOI:** 10.7759/cureus.29665

**Published:** 2022-09-27

**Authors:** Gurtej Bajaj, Warren A Southerland, Ignacio Badiola, Russell Bell

**Affiliations:** 1 Physical Medicine and Rehabilitation, University of Pennsylvania, Philadelphia, USA; 2 Pain Medicine, Minivasive Pain & Orthopedics, Houston, USA; 3 Anesthesiology, University of Pennsylvania, Philadelphia, USA

**Keywords:** pain medicine, case report, epidural blood patch, post dural puncture headache, spinal cord stimulator trail leads

## Abstract

Spinal cord stimulators (SCS) have been an invaluable resource in treating chronic pain pathologies such as failed back surgery syndrome, complex regional pain syndrome, neuropathic pain, and leg ischemia. Post-dural puncture headaches (PDPH) are a common phenomenon that happens when the dura is compromised. It has been seen with permanent SCS placement, but less commonly reported with SCS trail leads. We present a case of a patient who developed PDPH symptoms, not after initial trial leads placement but upon their removal. This case not only illustrates that dural compromise can occur when the placement of the leads is correct with confirmatory imaging, but also the leads themselves can contribute to masking the defect.

## Introduction

Since its inception in 1967, spinal cord stimulators (SCS) have been an important resource in treating chronic pain pathologies stemming from failed back surgery syndrome, complex regional pain syndrome, neuropathic pain, and leg ischemia [1]. The incidence of complications with SCS has been reported in 30-40% of cases, ranging from hardware complications to infections [2]. Post-dural puncture headaches (PDPH) are a common phenomenon that can be seen in neuraxial procedures due to dural compromise. Such a complication has been recorded in literature with SCS placement; however, what has been less documented still is the development of PDPH symptoms after SCS trial lead removal without noted symptomatology during trail SCS lead placement with improvement in symptoms after an epidural blood patch (EBP).

## Case presentation

A 56-year-old female with history of fibromyalgia, L4-L5 and S1 facet arthropathy, and cervical spondylosis presented in early 2021 to the pain clinic for bilateral neck pain and left buttock pain. She noted that her low back and buttock pain started in 2015 that radiated down to her left lateral foot without noted weakness in her lower extremities. Examination and history led to bilateral sacroiliac injections and piriformis injections without any relief in her discomfort. Electromyography was performed that did not show any nerve condition or muscle activation abnormalities. She was referred to pain psychology in November 2021 for cognitive behavioral therapy to supplement the pharmacological avenue of treating her pain. She was rotated through various muscle relaxers without improvement in pain control. She was unable to tolerate gabapentin and pregabalin but continued a stable dose of her duloxetine for treatment of her fibromyalgia.

Given the lack of improvement in her pain, she underwent a spinal cord stimulator trial in June 2022. The procedure was performed without any complications or observed signs of dural puncture. Post-operatively, she reported a transient headache when waking from anesthesia that resolved quickly. On her follow-up appointment one week later, she noted a 50% reduction in her lower back and extremity pain with improvement in functionality. She had no headaches during the week-long trial. Her trial SCS leads were removed with plans for permanent SCS placement. The following day the patient reported frontal headaches that were worse with sitting and standing, improved by laying supine, nausea and vomiting, and photophobia. She was advised to treat her headache with conservative measures, such as fluids, caffeine, and butalbital-acetaminophen-caffeine. The patient called the clinic two days later reporting her symptoms did not improve and continued to be tremendously bothersome, resulting in her being bedbound. Due to the concern for PDPH and lack of response to conservative measures, she was brought to the clinic for an EBP. The procedure was performed without complications. The epidural space was accessed at the L3-L4 space and 12 mL of autologous blood was injected over a two-minute period. Upon completion of her outpatient EBP, she noted a complete resolution of her symptoms by the time she left the pain clinic. The patient was able to return to her normal daily activities shortly after the procedure and committed to a permanent spinal cord stimulator placement in 2022.

## Discussion

One retrospective study demonstrated that the frequency of PDPH from SCS implantation (both trial and permanent) was reported to be 0.81% per lead placement [1]. Another large, retrospective study assessed the incidence of inadvertent dural puncture in over 90,000 patients undergoing percutaneous SCS and found that the incidence of dural puncture was 0.48%. The patients more likely to experience such complications are those similarly found in epidemiological studies of PDPH after neuroaxial procedures, such as younger age, female, and prior history of dural compromise [1,3]. Other factors associated with PDPH include low BMI and utility of 14-gauge non-pencil point needle [4]. While PDPH usually lasts two weeks or less and is self-resolving, its presence can dramatically affect patients’ livelihoods [5]. Treatment for PDPH ranges from conservative (fluids, non-steroidal anti-inflammatory drugs (NSAIDs), caffeine) to invasive (EBP), which can be pursued earlier if symptomatology is dramatic or conservative measures are not effective [6]. Eldrige et al. describe two patients who had PDPH symptoms soon after permanent SCS placement. Both patients failed conservative management but had complete resolution of their symptoms upon EBP administration [7]. Hussain et al. quantified in their retrospective report that therapeutic EBP was performed in about 64% of patients who had PDPH symptoms after SCS placement, with a median administration of four days. The proportion of those who were recommended for this intervention just days after symptomatology points towards not only the morbidity caused by PDPH but in addition the reliable results that can be seen with an EBP.

Studies show conflicting outcomes of prophylactic intraoperative EBP for treating PDPH. The Neurostimulation Appropriateness Consensus Committee guidelines rate intraoperative blood patch as evidence level III and recommendation C [8]. One survey study discovered that when dural compromise was seen during the placement of trail and permanent SCS, about 57% and 62%, respectively, of physicians would continue with placement but on a different level [9]. The study infers that when the procedure was not aborted by the interventionalist, an EBP was performed intraoperatively to facilitate closure of the known dural defect.

The patient presented in this report provides a unique scenario, in that the patient experienced PDPH symptoms not after the SCS trial and, presumed, original insult, but instead after the removal of the temporary leads, which, to the best of our knowledge, has not been previously documented. At the time of trial lead placement, there was no indication of a dural puncture, and the patient did not have symptoms of PDPH post-operatively until removal of the leads. However, it is believed that dural puncture did occur on lead placement and it was the leads themselves that masked the puncture. Just as a physical barrier is created by an EBP to clot off the site of a CSF leak, we believe the leads were providing a similar function by creating a barrier to disallow CSF leakage. Secondly, the possibility of the leads abutting the thecal sac could have caused increased or sustainment of normal CSF pressures to the cranium. Both explanations could have occurred simultaneously, but upon removal of the leads, the defect was exposed with resultant PDPH symptomatology.

## Conclusions

This case report highlights the possibility of a dural defect with trail SCS placement that reveals itself after trail lead removal, even when the initial placement was without signs of dural puncture. While uncommonly reported, proceduralists should be attentive to the possibility of the scenario described in this case report so as to avoid an unnecessary medical workup. In addition, EBP is an effective, efficient approach to resolve the complication and allow a patient to progress toward permanent SCS placement to address their chronic pain.
